# Genome‐wide functional analysis of hot pepper immune receptors reveals an autonomous NLR clade in seed plants

**DOI:** 10.1111/nph.16878

**Published:** 2020-11-16

**Authors:** Hye‐Young Lee, Hyunggon Mang, Eunhye Choi, Ye‐Eun Seo, Myung‐Shin Kim, Soohyun Oh, Saet‐Byul Kim, Doil Choi

**Affiliations:** ^1^ Plant Immunity Research Center Seoul National University Seoul 08826 Korea; ^2^ Department of Plant Science, Plant Genomics and Breeding Institute Research Institute for Agriculture and Life Sciences Seoul National University Seoul 08826 Korea

**Keywords:** *Capsicum annuum*, cell death, coiled‐coil domain, plant NLR, singleton NLR

## Abstract

- Plants possess hundreds of intracellular immune receptors encoding nucleotide‐binding domain leucine‐rich repeat (NLR) proteins. Full‐length NLRs or a specific domain of NLRs often induce plant cell death in the absence of pathogen infection.
- In this study we used genome‐wide transient expression analysis to identify a group of NLRs (ANLs; ancient and autonomous NLRs) carrying autoactive coiled‐coil (CC^A^) domains in pepper (*Capsicum annuum*). CC^A^‐mediated cell death mimics hypersensitive cell death triggered by the interaction between NLRs and pathogen effectors.
- Sequence alignment and mutagenesis analyses revealed that the intact α1 helix of CC^A^s is critical for both CC^A^‐ and ANL‐mediated cell death. Cell death induced by CC^A^s does not require *NRG1*/*ADR1* or *NRC* type helper NLRs, suggesting ANLs may function as singleton NLRs. We also found that CC^A^s localize to the plasma membrane, as demonstrated for *Arabidopsis* singleton NLR ZAR1. Extended studies revealed that autoactive CC^A^s are well conserved in other *Solanaceae* plants as well as in rice, a monocot plant. Further phylogenetic analyses revealed that ANLs are present in all tested seed plants (spermatophytes).
- Our study not only uncovers the autonomous NLR clade in plants but also provides powerful resources for dissecting the underlying molecular mechanism of NLR‐mediated cell death in plants.

## Introduction

Plants have evolved multiple immune receptors for the activation of defense responses to pathogen attack (Dodds & Rathjen, 2010; Dangl *et al*., 2013). In plant cells, nucleotide‐binding domain leucine‐rich repeat (NLR) proteins monitor pathogen invasion via direct or indirect sensing of effectors derived from pathogens (Dangl *et al*., 2013). After recognition, activated NLRs undergo a conformational change and initiate immune signaling (Moffett *et al*., 2002; Wang *et al*., 2019b). Following NLR‐mediated immune activation, a variety of defense responses are activated in infected tissues, including calcium flux, production of reactive oxygen species (ROS), activation of mitogen‐activated protein kinases, biosynthesis of antimicrobial secondary metabolites, signaling by plant defense hormones, and upregulation of a subset of defense‐related genes, which is often associated with the hypersensitive response (HR), a type of programmed cell death (Bonardi *et al*., 2012; Meng & Zhang, 2013).

Genome‐wide analyses have revealed that the NLR repertoire is diverse in terms of both quality and quantity among plant species, forming distinct phylogenetic clusters (Jacob *et al*., 2013). Although considerable effort has been expended over the last few decades to elucidate the mechanism of NLR‐mediated resistance, the question of how multiple NLR clades function in plant immune responses remains largely unanswered.

Plant NLRs were recently classified into three different categories based on mode of action: singleton, pair, and network (Adachi *et al*., 2019b). Singleton NLRs function as a single genetic unit for both sensing and signaling (Catanzariti *et al*., 2010; Krasileva *et al*., 2010; Adachi *et al*., 2019b). These NLRs have the potential to confer resistance when expressed in heterologous plants, even in taxonomically distant plants, as no other NLRs are required for their full activity (Saur *et al*., 2019). In addition, most singleton NLRs possess a signaling domain that induces visible cell death in heterologous plants.

Paired NLRs function such that one serves as a ‘sensor’ to detect pathogen effectors, while the other functions as an immune signaling ‘executor’ (Rodriguez‐Moreno *et al*., 2017). The typical NLR pairs are RESISTANCE TO *RALSTONIA SOLANACEARUM* 1 (*RRS1*)/RESISTANCE TO *PSEUDOMONAS SYRINGAE* 4 (*RPS4*) in Arabidopsis and RESISTANCE GENE ANALOG 5 (*RGA5*)/*RGA4* in rice (*Oryza sativa*). These genes are genetically linked and oriented head‐to‐head so that they can share a promoter for simultaneous regulation of transcription. Ectopic expression of executor NLR in NLR pairs often results in cell death without its effector. This autoactivity is inhibited by co‐expression of paired sensor NLRs, indicating that sensor NLRs may also function as negative regulators of executor NLRs in the absence of pathogen infection (Jin *et al*., 2002; Zhang *et al*., 2004). Sensor NLRs in typical NLR pairs generally have an additional integrated domain that is unusual and functions in recognizing effector proteins; this domain then relieves the regulation of the executor NLR, resulting in activation of immune responses. Although *in silico* analyses have revealed that NLRs with integrated domains are common in higher plants, functional NLR pairs have been discovered in only a few plant species (Saucet *et al*., 2015).

A more complex NLR model was reported as an ‘NLR network’, primarily in Solanaceous plants. Helper NLRs known as *NLR‐REQUIRED FOR CELL DEATH* (*NRC*) are associated with phylogenetically linked sensor NLRs and exhibit functional redundancy for conferring resistance to a diverse array of pathogens (Wu *et al*., 2017).

NLR proteins are generally composed of three major domains: a variable N‐terminus, a central nucleotide‐binding (NB‐ARC) domain, and a C‐terminal leucine‐rich repeat (LRR) domain. In general, NLRs are divided into two major groups based on the N‐terminal domain (NTD; Meyers *et al*., 2003; Jones & Dangl, 2006). The NLRs with a Toll/interleukin‐1 receptor‐like (TIR) domain at the N‐terminus are referred as TNLs, and those with coiled‐coil (CC) structure are referred to as CNLs (Jones & Dangl, 2006). One group of CNLs carrying CC domains resembling Arabidopsis resistance protein RPW8 is considered to represent a distinct subclass, RPW8‐type CNLs (RNL), based on their function in the downstream signaling (Peart *et al*., 2005; Bonardi *et al*., 2011; Collier *et al*., 2011). RNLs are highly conserved in plant species, and they are necessary for the functioning of other NLRs (Collier *et al*., 2011). One particular RNL gene, *N REQUIREMENT GENE 1* (*NRG1*), is specific to TNL‐mediated immunity and partially contributes to the signaling of some CNLs (Castel *et al*., 2019). Other RNL, known as *Activated Disease Resistance 1* (*ADR1*), also associate with various TNLs and the signaling of some CNLs (Bonardi *et al*., 2011).

Structural analyses of NLRs have revealed that the NTD plays multiple regulatory roles. For a number of NLRs, the NTD interacts with host target proteins manipulated by effector proteins (Mucyn *et al*., 2006; Ade *et al*., 2007; Burch‐Smith *et al*., 2007; Sacco *et al*., 2007). Interactions between homotypic NTDs contribute to the formation of higher‐order complexes of NLRs upon activation (Mestre & Baulcombe, 2006; Bernoux *et al*., 2011; Maekawa *et al*., 2011). Moreover, the NTD is also involved in the transduction of cell death signals. Overexpression of the NTD of some NLRs is sufficient to trigger cell death without cognate effector proteins (Peart *et al*., 2005; Bernoux *et al*., 2011; Bonardi *et al*., 2011; Collier *et al*., 2011; Maekawa *et al*., 2011; Casey *et al*., 2016; Hamel *et al*., 2016). However, the mechanism by which most CNLs are activated and trigger cell death still remains unknown.

Recent reports described the reconstitution of inactive and active complexes of an Arabidopsis CNL, HOPZ‐ACTIVATED RESISTANCE 1 (ZAR1), with receptor‐like cytoplasmic kinases, using structural and biochemical approaches (Wang *et al*., 2019a; Wang *et al*., 2019b). Cryo‐electron microscopy structural analyses have demonstrated that activated ZAR1 forms a wheel‐like pentamer resistosome and then undergoes a conformational change that exposes a funnel‐shaped structure formed by the N‐terminal α1 helix of the CC domain. These results suggest that the exposed α1 helix of the ZAR1 resistosome mediates cell death by translocating into and perturbing the integrity of the plasma membrane (PM) (Wang *et al*., 2019a). However, whether the ZAR1 model sufficiently explains CC‐induced cell death remains to be elucidated.

Solanaceous plants belong to a large family consisting of over 3000 species, including important crops such as potato (*Solanum tuberosum*), tomato (*Solanum lycopersicum*), and pepper (*Capsicum annuum*) (Chiarini & Bernardello, 2006). Previous comparative analyses of NLRs across Solanaceae genomes revealed that the NLR gene family can be classified into one TNL and 14 CNL subgroups (Seo *et al*., 2016). Pepper CNL‐Group 10 (G10) contains 34 genes, including the known disease‐resistance (R) genes *Pvr4* and *Tsw*, the products of which confer resistance to potyviruses such as *Potato virus* *Y* and *Tomato spotted wilt virus* via recognition of a viral effector, respectively (Kim *et al*., 2015, 2017). Structural domain analyses have revealed that the CC domain of Pvr4 is sufficient to activate cell death in the absence of viral effector NIb (Kim *et al*., 2018).

In this study, we conducted a genome‐wide screening in *Nicotiana benthamiana* and report that the CC domains of G10‐NLRs (hereafter termed ANLs; ancient and autonomous NLRs) in pepper specifically induce HR‐like cell death. Autoactive ANLs and CC domains of ANLs (CC^A^)‐mediated cell death was associated with immune responses. CC^A^s were found to localize to the PM, where the α1 helix of these domains plays a critical role in mediating cell death. Cell death induced by autoactive ANLs and CC^A^s was not compromised in *NRG1*/*ADR1* or *NRC* type helper NLR‐silenced plants, suggesting that these helper NLRs may be not required for signaling associated with ANL‐mediated cell death. Surprisingly, ANLs were found to be well conserved in seed plants. CC^A^s in other Solanaceae plants, as well as a monocot plant, rice, induced cell death in *N. benthamiana*. We propose that ANLs represent a novel singleton NLR clade and that CC^A^s could serve as powerful resources for elucidating the underlying molecular mechanism of NLR‐mediated plant cell death.

## Materials and Methods

### Plant materials and growth conditions

*Nicotiana benthamiana* plants were grown in horticultural bed soil (Biogreen, Seoul, Korea) in a well‐maintained chamber under the following conditions: 16 h : 8 h, light : dark photoperiod, temperature of 25°C, photosynthetic photon flux density of 80–100 µmol m^−2^ s^−1^ and relative humidity of 70%. Four‐week‐old plants were used for transient overexpression. Foliage leaves of 2‐wk‐old plants were inoculated with *Agrobacterium* for virus‐induced gene silencing (VIGS).

### Identification of NLRs and phylogenetic tree analysis

Annotated protein sequences of *Capsicum annuum* L. v.1.55 (Kim *et al*., 2014), *Nicotiana tabacum* L. Nitab v.4.5 (Edwards *et al*., 2017), *Oryza sativa* L. Rgap 7.0 (Kawahara *et al*., 2013), *Picea abies* (L.) H. Karst. v.1.0 (Nystedt *et al*., 2013), *Piper nigrum* L. v.1.0 (Hu *et al*., 2019), *Selaginella moellendorffii* Hieron v.1.0 (Banks *et al*., 2011), *Solanum lycopersicum* L. Itag 2.4 (Sato *et al*., 2012), *Solanum tuberosum* L. PGSC 3.4 (Xu *et al*., 2011) and known NLR genes from GenBank and the Plant Resistance Genes Database v.3.0 (Osuna‐Cruz *et al*., 2018) were used in this study. NLR identification and classification methods were based on a previous study (Seo *et al*., 2016) with some modifications. To identify NLRs containing the NB‐ARC domain (PF00931), interproscan v.5.22‐61 (Jones *et al*., 2014) was run with the default parameters. Subsequently, these NLRs were scanned with mast in meme v.4.9.1 (Bailey *et al*., 2009) using NB‐ARC motif information from nlr‐parser v.1.0 (Jupe *et al*., 2012; Steuernagel *et al*., 2015). NB‐ARC domains with at least three of the four major motifs (P‐loop, GLPL, Kinase2 and MHDV) in order and a length of at least 160 residues were selected as intact NB‐ARC domains and aligned using mafft v.7.407 (–maxiterate 1000 –globalpair) (Katoh & Standley, 2013). Positions containing gaps of ≥ 90% in multiple sequence alignment were trimmed using trimal v.1.4.rev22 (Capella‐Gutierrez *et al*., 2009). Phylogenetic relationships were reconstructed based on the maximum‐likelihood method using iq‐tree v.1.6.12 (Nguyen *et al*., 2015) with 1000 ultrafast bootstrap approximation (UFBoot) (‐bb 1000 ‐safe) (Hoang *et al*., 2018). Substitution models were selected using Modelfinder (Kalyaanamoorthy *et al*., 2017) in iq‐tree. The best‐fit model was JTT + F+R9. NLR groups were assigned based on known NLR genes, UFBoot value > 90% and previously assigned group information (Seo *et al*., 2016). Phylogenetic trees and heatmaps of the proportion of intact NB‐ARC domains for each NLR group in plant species were plotted using ggtree v.1.6.11 (Yu *et al*., 2017, 2018). The plant species tree was generated using timetree (Kumar *et al*., 2017). For motif analysis, CC domains of the pepper NLRs were scanned to find the EDVID motif (motif_16 from nlr‐parser) and MADA motif (Steuernagel *et al*., 2015; Adachi *et al*., 2019a). The parameters for MADA‐HMM were as per a recent study (Adachi *et al*., 2019a) using hmmsearch (–max option and a HMM score cut‐off of 10.0) in hmmer v.3.1b2 (Eddy, 2011).

### Plasmid construction

The fragments of pepper NLRs were amplified from genomic DNA of *C. annuum* CM334 based on the pepper reference annotation v.1.55 (Kim *et al*., 2014). To amplify target genes, primers were specifically designed within 1 kb of the 5′‐ or 3′‐UTR for each gene. Amplicons were cloned into the pCAMBIA2300‐LIC vector containing a cauliflower mosaic virus 35S promoter and nopaline synthase terminator using the ligation‐independent cloning (LIC) method (Oh *et al*., 2010; Kim *et al*., 2017). The N‐terminal domain of NLR was defined as spanning from methionine to just before the P‐loop motif of the NB‐ARC domain. Amplified NTD fragments were cloned into the pCAMBIA2300‐LIC or pCAMBIA2300‐3xFLAG‐LIC vector in the same manner as full‐length NLRs. For cloning of CC domains from other plant species, the fragments were amplified from genomic DNA of rice (*O. sativa* cv Nakdong) and tobacco (*N. tabacum* cv Xanthi) or cDNA of tomato (*S. lycopersicum* cv Heinz) and potato (*S. tuberosum*). To silence *NRC2/3/4*, the partial fragments of *NbNRC2*, *NbNRC3* and *NbNRC4* were combined by overlap polymerase chain reaction (PCR), and the overlapped fragment was cloned into the pTRV2‐LIC vector. For RNL silencing, short fragments of *NbNRG1* and *NbADR1* were combined by overlap PCR, and the overlapped fragment was cloned into the TRV2‐LIC vector. The primers used for PCR in this study are listed in Supporting Information Dataset S1.

### Site‐directed and motif‐swap mutagenesis

N‐terminal motif‐swap and site‐directed mutants were generated using specific primers carrying the desired mutations. The amplified fragments for tag fusion proteins were cloned into the pCAMBIA2300‐3xFLAG‐LIC or pCAMBIA2300‐eGFP‐LIC vector using a LIC method.

### *Agrobacterium*‐mediated transient overexpression in *N. benthamiana*

*Agrobacterium tumefaciens* GV3101 strains carrying the various constructs were prepared for transient overexpression. Bacteria were grown overnight at 28°C in Luria‐Bertani (LB) medium supplemented with kanamycin (50 μg ml^−1^) and rifampicin (50 μg ml^−1^). The cells were then pelleted and resuspended in infiltration buffer (10 mM 2‐(*N*‐morpholino)ethanesulfonic acid (MES; pH 5.6) and 10 mM magnesium chloride (MgCl_2_) with 150 μM acetosyringone) at an optical density at 600 nm (OD_600_) of 0.3 for CC domains or 0.6 for full‐length NLRs and R genes. To screen for autoactive CC domains in ANLs from tomato, tobacco, potato, and rice, CC^A^s were co‐expressed with the gene‐silencing suppressor p19 (final OD_600_ = 0.25). Agrobacterial suspensions were applied to infiltrate the abaxial leaves of 4‐wk‐old *N. benthamiana* plants using a needleless syringe. Cell death was detected using a fluorescence‐labeled organism bioimaging instrument system (Neoscience, Suwon, Korea). The degree of cell death was reported as quantum yield (*F*_v_/*F*_m_) using a closed FluorCam (Photon Systems Instruments, Czech Republic) (Jones *et al*., 2001).

### Disease‐resistance assay

For *Potato virus X–*green fluorescent protein (PVX:GFP) co‐expression, *A. tumefaciens* GV3101 carrying the PVX:GFP‐expressing vector was co‐infiltrated with CC^A^, ANLs and Pvr4/NIb. At 30 h post‐infiltration (hpi), the intensity of GFP fluorescence was measured using a closed FluorCam system (Photon Systems Instruments, Drasov, Czech Republic) with a GFP filter to quantify PVX replication.

### Virus‐induced gene silencing

Virus‐induced gene silencing was performed as described by Liu *et al*. (2002). Suspensions of pTRV1 and pTRV2 carrying the target gene fragment were mixed in a 1 : 1 ratio in infiltration buffer at a final OD_600_ of 0.15. Two leaves of 2‐wk‐old *N. benthamiana* seedlings were infiltrated with the *Agrobacterium* suspension mixture. Three weeks later, the upper leaves were collected to confirm silencing of the target gene and to be used for subsequent experiments.

### Gene expression analyses

**Fig. 1:**
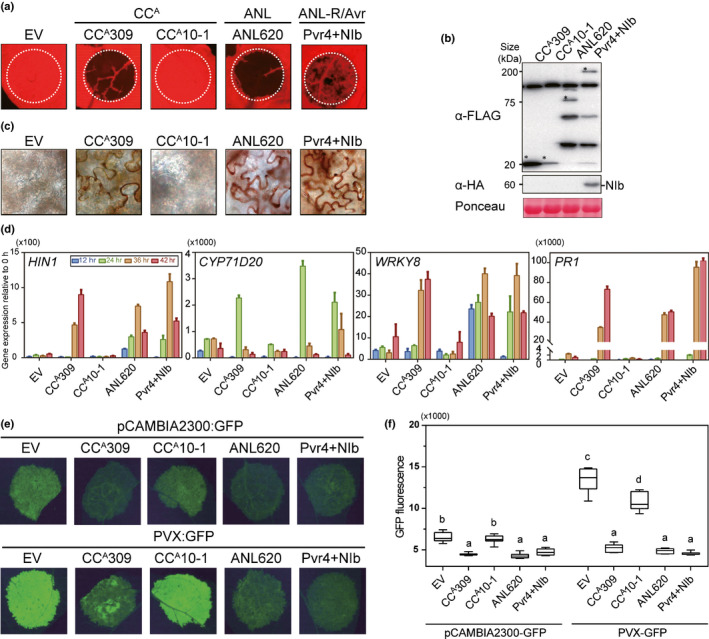
Autoactive ANLand CC^A^ trigger defense‐related cell death. (a) CC^A^309, ANL620 and Pvr4 with its cognate effector NIb induce cell death, but CC^A^10‐1 does not. An empty vector (EV) was used as a negative control. The leaves of 4‐wk‐old *Nicotiana benthamiana* were infiltrated with agrobacteria harboring each construct. Images were taken 3 d post infiltration (dpi). (b) Protein accumulation was confirmed by immunoblot analysis. Equal protein loading was confirmed by staining membranes with Ponceau S. Asterisks indicate the expected protein bands. (c) H_2_O_2_ accumulated in CC^A^309‐, ANL620‐ and Pvr4/NIb‐infiltrated leaves but not CC^A^10‐1‐or EV‐infiltrated leaves. Leaves were stained with 3,3′‐diaminobenzidine hydrochloride (DAB) at 2 dpi; *n* = 3. (d) Expression of defense‐related genes *HIN1*, *CYP71D20*, *WRKY8* and *PR1* was dramatically elevated by CC^A^309, ANL620, and Pvr4/NIb. Four‐week‐old *N. benthamiana* leaves infiltrated with EV, CC^A^309, CC^A^10‐1, ANL620 and Pvr4/NIb were collected at various time points. The data are shown as mean values ± SD (*n* = 3).(e, f) Enhanced resistance to *Potato virus X* mediated by CC^A^309, ANL620 and Pvr4/NIb. pCAMBIA2300:GFP or PVX:GFP were co‐infiltrated with agrobacteria carrying EV, CC^A^309, CC^A^10‐1, ANL620 or Pvr4/NIb. (f) Accumulation of green fluorescent protein (GFP) was visualized at 30 hpi. Green fluorescent protein fluorescence emission was quantified using a FluorCam system with a GFP filter. Letters indicate groupings by statistical difference, as determined by one‐way ANOVA with Sidak's multiple comparisons test. Error bars indicate SD. The above experiments were repeated twice with eight biological replicates.

To evaluate transcriptional upregulation of defense‐related genes, RNA was extracted at indicated time points in Fig. 1(d) from leaf tissues infiltrated with Agrobacteria containing a full‐length NLR or an NTD. Agrobacteria containing an empty vector were used as a negative control. Total RNA was extracted using TRIzol reagent (MRC, Cincinnati, OH, USA), and cDNA was synthesized using Superscript II (Invitrogen, Carlsbad, CA, USA). Gene‐specific primers were used for quantitative reverse transcription (RT) polymerase chain reaction (qRT‐PCR) at 95°C for 5 min, followed by 40 cycles of denaturation at 95°C for 15 s and 55°C for 1 min. Quantitative RT‐PCR was performed using a CFX96 real‐time PCR instrument (Bio‐Rad, Hercules, CA, USA) with SsoAdvanced universal SYBR green supermix (Bio‐Rad). Nucleotide sequences of all primers used in this study are listed in Dataset S1. Gene transcript levels were normalized to that of the elongation factor gene, *NbEF1‐α*.

### Confocal laser scanning microscopy

Cells expressing GFP‐tagged proteins were examined using an SP8X confocal microscope (Leica Microsystems, Wetzlar, Germany) with a ×40/1.0 water‐dipping objective. Cells were imaged using 488‐nm excitation and detection of emission from 500–530 nm. Simultaneous excitation of GFP and PM marker dye FM4‐64 (Invitrogen), was performed using 488‐nm excitation and detection of emission signals at 500–530 nm and 600–650 nm for GFP and FM4‐64, respectively.

### DAB (3,3′‐diaminobenzidine hydrochloride) staining

Accumulation of H_2_O_2_ was monitored by DAB staining. Leaves were detached and incubated in DAB‐HCl solution (1 mg ml^−1^, pH 3.8) overnight at 25°C in the dark. After staining, the leaves were soaked in 95% ethanol to remove chlorophyll.

### Immunoblot analysis

Leaf tissue (50 mg) of *N. benthamiana* was ground into fine powder in liquid nitrogen and homogenized in 200 μl of extraction buffer (50 mM Tris‐HCl (pH 7.5, 100 mM NaCl, 1 mM ethylenediaminetetraacetic acid (EDTA), 1% (v/v) octylphenoxy poly(ethyleneoxy)ethanol (IGEPAL CA‐630), 10% glycerol, 5 mM dithiothreitol (DTT), 1 mM sodium fluoride (NaF), 1 mM phenylmethylsulfonyl fluoride (PMSF), and 1 × EDTA‐free protease inhibitor cocktail (Takara, Shiga, Japan)). The lysates were cleared by centrifugation at 12 000 ***g*** for 10 min at 4°C. Samples were loaded onto 8% or 10% sodium dodecyl sulfate–polyacrylamide gel electrophoresis (SDS‐PAGE) gel, and proteins were detected after blotting with anti‐FLAG M2 antibody (Sigma, St Louis, MO, USA) (1 : 10 000 dilution) or anti‐HA antibody (Abcam, Cambridge, UK) (1 : 5000 dilution).

### Quantification and statistical analyses

Statistical analyses and graph generation were performed using Prism7 software (GraphPad, San Diego, CA, USA). Statistical comparisons between different samples were carried out by one‐way or two‐way analysis of variance (ANOVA) with Sidak's multiple comparisons or Dunnett’s multiple comparisons tests.

## Results

### The CC domains of pepper ANLs induce cell death in *N. benthamiana*

In a previous study, intact pepper NLRs were identified and assigned into 15 groups, consisting of 1 TNL group and 14 CNL groups (Seo *et al*., 2016). To identify autoactive NLRs on a genome‐wide scale, genomic fragments of 436 intact pepper NLRs were cloned and transiently expressed in *N. benthamiana* leaves via agroinfiltration (Kim *et al*., 2014). The sequences of all inserts were confirmed by DNA sequencing analysis (Dataset S2). Among the 436 tested NLRs, only 15 (3.4%) triggered cell death (Table 1). Among the 15 NLRs, 6 were TNLs and the others were distributed into five CNL groups: CNL‐G5, G9, G10 (ANL), G11 and NG. To explore whether these genes possibly function as executor NLRs in typical paired sensor NLRs, we examined the upstream flanking genes of the autoactive NLRs using pepper chromosome pseudomolecules, as conventional NLR pairs are genetically linked in a head‐to‐head orientation (Sukarta *et al*., 2016; Kim *et al*., 2017). Although 9 of 15 autoactive NLRs had an NLR‐type gene in the neighboring region, none were arranged in a head‐to‐head orientation (Table S1). Furthermore, the sequences of these neighboring NLR genes did not encode an integrated domain, which is a typical feature of sensors of paired NLRs. These results suggest that the conventional paired NLRs, such as Arabidopsis *RPS4*/*RRS1* and rice *RGA4*/*RGA5*, probably do not exist in pepper.

**Table 1:** Screening of autoactive pepper NLRs and N‐terminal domains in *Nicotiana benthamiana*.

Group	No. assigned NLR	Full‐length NLR		N‐terminal domain (NTD)	
		No. tested NLR	No. autoactive NLR (%)	No. tested NTD	No. autoactive NTD (%)
TNL	54	49	6 (12.2)	16	2 (12.5)
CNL‐G1	75	63	0 (0)	10	0 (0)
CNL‐G2	91	81	0 (0)	13	0 (0)
CNL‐G3	23	23	0 (0)	8	0 (0)
CNL‐G4	32	31	0 (0)	9	0 (0)
CNL‐G5	12	12	1(8.3)	4	0 (0)
CNL‐G6	26	25	0 (0)	5	0 (0)
CNL‐G7	18	16	0 (0)	7	0 (0)
CNL‐G8	14	14	0 (0)	6	0 (0)
CNL‐G9	48	48	1 (2.1)	9	0 (0)
CNL‐G10 (ANL)	34	33	3 (9.1)	24	17 (70.8)
CNL‐G11	7	7	1 (14.3)	5	1 (20)
CNL‐G12	9	8	0 (0)	5	0
CNL‐G13	1	1	0 (0)	1	0
CNL‐RNL	3	3	0 (0)	3	2 (66.7)
CNL‐NG	22	22	3 (13.6)	5	0 (0)
Total	469	436	15	131	22

ANL, ancient and autonomous NLR; NLR, nucleotide‐binding domain leucine‐rich repeat; NG, nongroup – not assigned to a CNL group; NTD, N‐terminal domain of NLR.

Previous studies reported that the N‐terminal TIR or CC domains of a specific set of resistance proteins, mostly potential singleton NLRs, induce cell death when overexpressed alone in plants (Collier *et al*., 2011; Sato *et al*., 2012; Baudin *et al*., 2017), suggesting that NLR proteins often trigger immune signaling via the NTD. To identify NLRs carrying an autoactive NTD in pepper, 131 representative NTDs, including those of autoactive full‐length NLRs, were cloned and transiently overexpressed in *N. benthamiana* in the same manner as the full‐length NLR. The NTDs tested for each group were chosen randomly and represented > 15% of all assigned NLRs from a subclade in a phylogenetic tree analysis (Table 1; Dataset S3). The NTD fragments were determined from the N‐terminus up to the predicted P‐loop in the NB‐ARC domain. As a result, only 22 NTDs (2 TIRs, 17 CCs in G10 (ANL), 1 CC in G11 and 2 CCs in RNLs) showed autoactivity. Of the 15 autoactive full‐length NLRs examined, only 2 TIR domains and 3 CC domains from autoactive NLRs induced visible cell death when expressed in *N. benthamiana* (Table S2).

Among the three RNLs, two CC domains from pepper homologs of NbNRG1 and NbADR1 triggered cell death (Tables 1, S2). In particular, compared with other groups, a significantly higher proportion (70.8%) of the CC domains from CNL‐G10 (ANLs) triggered cell death (Table 1; Fig. S1). Ancient and autonomous NLRs formed a distinct cluster in the phylogenetic tree that included several cloned functional R genes: *Pvr4* and *Tsw* in pepper and *RPS2*, *RPS5, SUT1* and *SUMM2* in Arabidopsis (Seo *et al*., 2016). Although they belong to a superclade of CNLs, ANLs are phylogenetically distinct from other CNL clades (Seo *et al*., 2016). Additional motif analysis revealed that the EDVID motif (Rairdan *et al*., 2008), a well‐conserved motif in the CC domain of functional CNLs was not predicted in pepper ANLs (Fig. S2a). Moreover, CC^A^s also do not possess the MADA motif that was recently reported to be conserved in CC domains in NRC‐type NLRs (G8) and *c.* 20% of CNLs (Fig. S2b) (Adachi *et al*., 2019a). Taken together, these data suggest that ANLs have distinct features not found in other CNLs and that they may play a unique role (or unique roles) in plant immunity.

### CC^A^‐induced cell death mimics R gene‐mediated HR

We next investigated whether cell death induced by autoactive ANLs or CC^A^s plays a role in R gene‐mediated defense responses against pathogens. An autoactive CC^A^, CC^A^309, a nonautoactive CC^A^, CC^A^10‐1, and an autoactive ANL, ANL620, were examined in this assay. A known ANL, *Pvr4,* a resistance gene for potyvirus, triggers HR cell death only if co‐expressed with its cognate Avr protein, NIb (Kim *et al*., 2017). Thus, Pvr4 with NIb was used as a positive control for R gene‐mediated cell death. Even though CC^A^309 and CC^A^10‐1 share high similarity (95.5% and 89.9% at the nucleotide and amino acid levels, respectively), they exhibited opposing phenotypes. Transient overexpression of CC^A^309 and ANL620, but not CC^A^10‐1, triggered visible death in *N. benthamiana* (Fig. 1a). All proteins were detected by immunoblot analysis, but the expression level of CC^A^10‐1 was much lower than that of CC^A^309 (Fig. 1b). To exclude the possibility that the nonfunctionality of CC^A^10‐1 is due to low protein accumulation, GFP was fused to CC^A^309 and CC^A^10‐1 at the C‐terminus. Although CC^A^309‐GFP and CC^A^10‐1‐GFP accumulated to similar levels, CC^A^10‐1‐GFP still did not trigger cell death, indicating that the nonautoactivity of CC^A^10‐1 is not attributed to low expression levels (Fig. S3).

Two days after agroinfiltration, we assessed hydrogen peroxide (H_2_O_2_) accumulation in the leaves using DAB staining. Hydrogen peroxide production was observed during cell death mediated by activated Pvr4 (Fig. 1c). As shown for Pvr4, CC^A^309 and ANL620, but not CC^A^10‐1, exhibited accumulation of H_2_O_2_. These results suggest that accumulation of reactive oxygen species, which is a defense response against pathogen infection, was accompanied by cell death induced by autoactive ANLs and CC^A^s.

To verify that ectopic expression of autoactive ANLs and CC^A^s mimics the activation of defense‐related genes, the CC^A^309‐ and ANL620‐mediated accumulation of transcripts for four defense‐related genes was monitored during cell death at various time points after agroinfiltration (Fig. 1d). Overexpression of CC^A^309 and ANL620, but not CC^A^10‐1, resulted in significantly higher transcription of several genes, including the HR cell death marker gene *Harpin‐induced 1* (*Hin1*), a transcription factor for physiological substrates of mitogen‐activated protein kinases, *WRKY8*, a cytochrome P450 involved in sesquiterpene phytoalexin biosynthesis, *CYP71D20*, and *Pathogenesis‐related gene 1* (*PR1*) (Gopalan *et al*., 1996; Glazebrook, 2005; Ishihama *et al*., 2011; Weitzel & Simonsen, 2015). These results indicate that cell death induced by autoactive ANLs and CC^A^s is also correlated with the accumulation of defense‐related gene transcripts.

Although ROS production and upregulation of defense‐related genes are typical immune responses, we cannot exclude the possibility that they also occur as a consequence of cell death. To verify that autoactive ANL and CC^A^ indeed trigger defense responses, we performed a co‐expression assay using a *Potato virus X* expressing GFP (PVX:GFP), which is able to establish compatible infection in *N. benthamiana* (Collier *et al*., 2011). We hypothesized that if autoactive CC^A^‐ or ANL‐mediated cell death is associated with activation of defense responses, replication of PVX:GFP would be restricted in co‐expressed cells. To avoid the possibility that cell death would affect the replication of PVX, the GFP signal was monitored before onset of the severe cell death phenotype (Fig. S4). Compared with the empty vector, Pvr4‐mediated cell death significantly reduced the abundance of PVX:GFP. Moreover, the GFP accumulation was also almost completely inhibited by expression of CC^A^309 and ANL620 (Fig. 1e,f). Taken together, this evidence shows that cell death induced by autoactive ANLs and CC^A^s functionally mimics R gene‐mediated HR cell death.

### The N‐terminal α1 helix is critical for autoactivity of CC^A^s

**Fig. 2:**
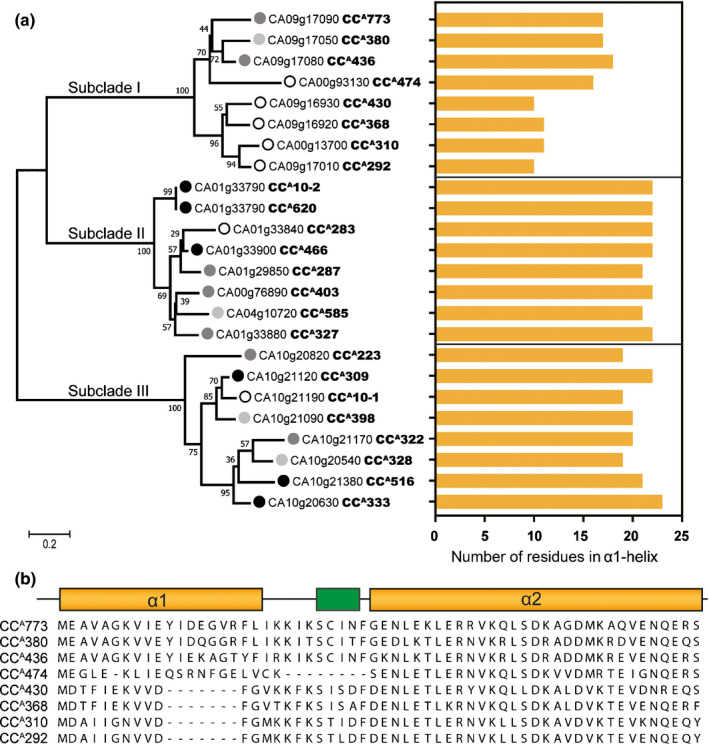
The first α‐helix region is critical for the autoactivity of CC^A^ domains. (a) Phylogenetic tree of CC^A^s (left) and length of the first α‐helix (right). The phylogenetic tree was constructed based on amino acid sequences of CC^A^‐domains. The maximum‐likelihood model was used, and bootstrap analysis was performed with 1000 replicates. The degree of cell death is represented as a grayscale circle to the left of the gene IDs (closed black circle, strong; dark gray circle, medium; light gray circle, weak cell death; open circle, no cell death). (b) Alignment of CC^A^s in subclade I and schematic representation of the secondary structure of CC domains in subclade I. Nonautoactive CC^A^s (CC^A^474, CC^A^292, CC^A^310, CC^A^368 and CC^A^430) are deficient in an intact first α‐helix. The α‐helix and β‐sheet are represented as yellow and green boxes, respectively.

We performed an alignment analysis of pepper CC^A^s to identify the conserved motif necessary for cell death activity. However, no conserved motif was found in any of the CC^A^s examined. A phylogenetic analysis based on amino acid sequences of pepper CC^A^s revealed that they are divided into three subclades, with 100% bootstrap confidence level (Fig. 2a). We found that most nonautoactive CC^A^s (shown by open circles in Fig. 2a) belong to subclade I. It was recently reported that the N‐terminal α1 helix of the ZAR1 CC domain is crucial for its cell death‐inducing activity (Wang *et al*., 2019a). Prediction of CC^A^ secondary structures using jpred4 (http://www.compbio.dundee.ac.uk/jpred4) revealed that nonautoactive CC^A^s (CC^A^292, CC^A^310, CC^A^368, and CC^A^430) in subclade I have a shorter predicted α1 helix than those of autoactive CC^A^s (Fig. 2a), and partial deletion of the α1 helix was found in nonautoactive CC^A^s (Fig. 2b). Although the α1 helix of CC^A^474 is longer than that of other nonautoactive CC^A^s, a deletion was found in the linker region between the α1 and α2 helices (Fig. 2a,b), suggesting that the deletion may disrupt formation of the critical structure for its activity. Taken together, these results suggest that the cell death‐inducing activity of nonautoactive CC^A^s in subclade I is associated with the deletion in the α1 helix.

**Fig. 3:**
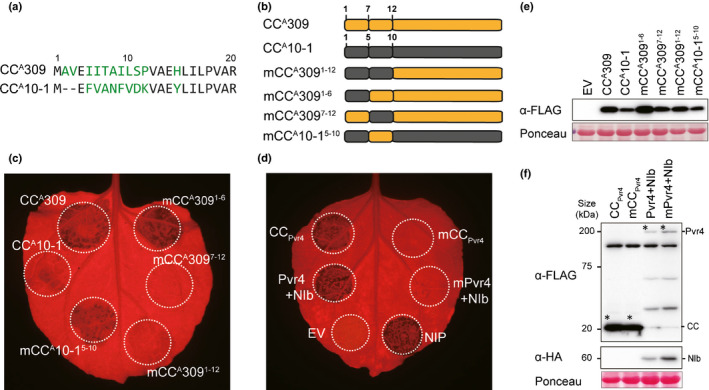
The N‐terminal α1 helix is critical for autoactivity. (a) Alignment of the N‐terminal region of CC^A^309 and CC^A^10‐1. Different residues are highlighted in green. (b) Schematic diagram of N‐terminal chimeras between CC^A^309 and CC^A^10‐1. (c) Cell death induced by the CC^A^309 and CC^A^10‐1 mutants described in (b). (d) Compromised cell death associated with defective α1 helix in Pvr4 and the CC domain of Pvr4 (CC_Pvr4_). *Phytophthora sojae* necrosis‐inducing protein (NIP) was used as a positive control for cell death. All swap and point mutants were transiently expressed in *Nicotiana benthamiana*, and cell death was visualized after 3 d post infiltration (dpi). (e, f) Protein accumulation as described in (d) was investigated using Western blotting analysis. Equal protein loading was confirmed by staining membranes with Ponceau S. The above experiments were repeated at least three times with similar results.

Although it has a sufficient length of α1 helix, CC^A^10‐1 is nonautoactive in subclade III. Sequence alignment with an autoactive CC^A^309 showed high sequence variation in the first α1 helix (Figs 3a, S5). Based on these observations, we focused on the function of the N‐terminus in cell death‐inducing activity. To confirm the function of this region for autoactivity, we generated CC^A^309 mutants in which amino acids 1–12, or half of them (i.e. 1–6 or 7–12), were substituted with those of CC^A^10‐1 (Fig. 3b) and expressed the mutants in *N. benthamiana*. Substitution of residues 7–12 (mCC^A^309^7–12^), as well as residues 1–12 (mCC^A^309^1–12^), but not residues 1–6 (mCC^A^309^1–6^), abolished cell death‐inducing activity (Fig. 3c). Conversely, mCC^A^10‐1^5–10^ carrying residues 7–12 of CC^A^309 exhibited slightly recovered activity, indicating that residues 7–12 of CC^A^309 are critical for autoactivity. To confirm that these residues are also critical for R gene‐mediated cell death, corresponding residues (residues 5–11) in CC_Pvr4_ and Pvr4 were mutated to those of CC^A^10‐1. As in CC^A^309, mutation of the N‐terminus disrupted the cell death‐inducing activity of CC_Pvr4_ and Pvr4 (Fig. 3d). Immunoblotting analysis revealed comparable expression levels of these protein fragments (Fig. 3e,f). Taken together, this evidence demonstrates that the first α helix region is crucial for CC^A^s and R gene‐mediated autoactivity.

### Cell death‐inducing activity of CC^A^309 is regulated by phosphorylation and Ca^2+^ influx

To uncover mechanistic insights into activation of cell death, we examined the subcellular distribution of CC^A^‐GFP in *N. benthamiana* using confocal microscopy and plasmolysis studies. GFP signals associated with CC^A^309 and CC^A^10‐1 were detected at the cell boundary and merged with a fluorescent dye specific to the PM (FM4‐64; Fig. S6). Moreover, other CC^A^s in subclades I and II also localized to the PM regardless of their cell death‐inducing activity (Fig. S7). These results suggest that nonautoactivity of CC^A^s is not due to mislocalization to other cellular compartments.

**Fig. 4:**
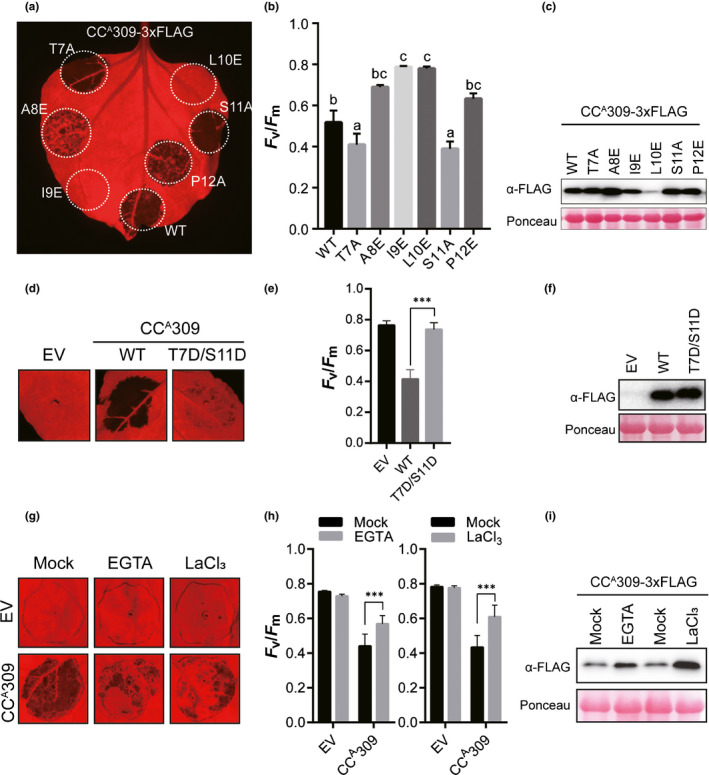
CC^A^309‐mediated cell death is associated with phosphorylation and calcium influx. (a) Cell death induced by point mutated CC^A^309 in *Nicotiana benthamiana*. Image taken at 3 d post infiltration (dpi). (b) Degree of cell death, quantified by quantum yield (*F*_v_/*F*_m_) using a closed FluorCam system. Letters indicate groupings by statistical difference, as determined by one‐way ANOVA with Sidak's multiple comparisons test. The data are shown as mean values ± SD. (c) Protein accumulation as described in (a). (d) Cell death induced by CC^A^309 T7D/S11D mutant. (e) Degree of cell death, quantified by quantum yield (*F*_v_/*F*_m_) using a closed FluorCam system. Significance was determined using one‐way ANOVA followed by Dunnett’s multiple comparisons test, with asterisks denoting statistically significant differences (***, *P* < 0.0001). Data are mean ± SD (*n* = 20). (f) Protein accumulation as described in (d). (g) Cell death induced by CC^A^309 was compromised by treatment with calcium chelating reagent EGTA and calcium channel blocker LaCl_3_. (h) Degree of cell death, quantified by quantum yield (*F*_v_/*F*_m_) using a closed FluorCam system. Significance was determined using one‐way ANOVA followed by Dunnett’s multiple comparisons test, with asterisks denoting statistically significant differences (***, *P* < 0.0001). Data are mean ± SD (*n* = 10). (i) Protein accumulation as described in (g).

Next, site‐directed mutagenesis using synthetic oligonucleotides was performed to change every residue in this region to alanine or glutamate. A8, I9, and L10 were substituted with negatively charged glutamate (E), and T7, S11 and 12P were substituted with alanine (A). The A8E mutation substantially reduced the cell death‐inducing activity, and the I9E and L10E mutations completely disrupted function (Fig. 4a,b), indicating that hydrophobic residues are essential for cell death‐inducing activity. All of the mutant proteins accumulated to similar levels (Fig. 4c), except for L10E, showing that the observed loss‐of‐function phenotype was not due to lack or destabilization of protein. Surprisingly, mutation of serine (S11A) and threonine (T7A), putative target residues of phosphorylation, triggered a high degree of cell death compared to that observed in wild‐type CC^A^309 (Fig. 4a,b). To confirm whetherthese putative phosphorylation residues in CC^A^309 are indeed important for its cell death‐inducing activity, a phospho‐mimic (S/T to D) mutant was generated. Despite expressing similar level of protein, a double phospho‐mimic mutant (T7D/S11D) showed clearly diminished cell death in *N. benthamiana* (Fig. 4d,e), suggesting that the autoactivity of CC^A^309 may be regulated by a phosphorylation/dephosphorylation event.

Hypersensitive response induced by PM‐localized NLR proteins often requires the influx of calcium ions into the cytoplasm from the extracellular space (Pike *et al*., 2005; Andersson *et al*., 2006). To determine whether CC^A^309‐mediated cell death also involves an influx of calcium ions, CC^A^309‐expressing leaves were treated with lanthanum(III) chloride (LaCl_3_), a typical blocker of Ca^2+^ influx, and egtazic acid (EGTA), a calcium chelating agent. Cell death induced by CC^A^309 was significantly compromised by both LaCl_3_ and EGTA without alteration of protein accumulation (Fig. 4g–i), indicating that an influx of Ca^2+^ is also required for CC^A^309‐induced cell death. Taken together, these findings suggest that CC^A^‐mediated cell death might be regulated by phosphorylation/dephosphorylation and requires the influx of calcium ions.

### Cell death mediated by CC^A^ is independent of RNL or NRC type helper NLRs

**Fig. 5:**
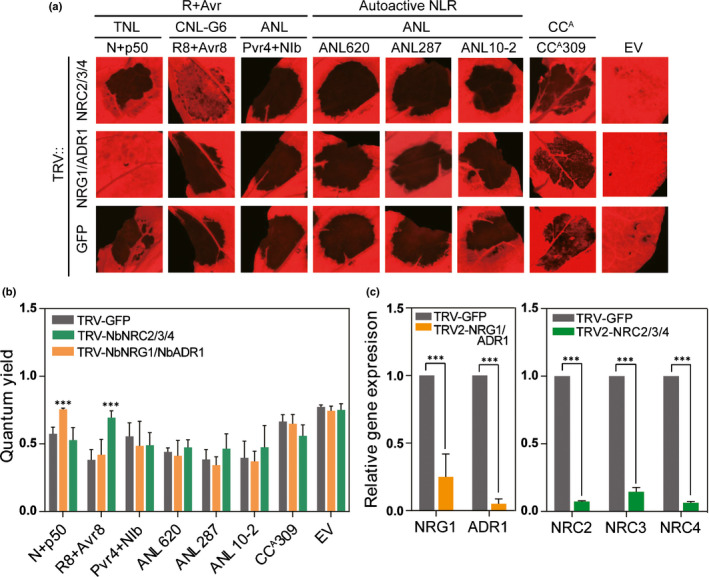
Cell death induced by autoactive CC^A^ or ANLs is independent of *NRG1*/*ADR1* or *NRC* type helper‐mediated pathways. (a) Cell death phenotype induced by autoactive ANLs and a CC^A^ in *NRG1*/*ADR1*‐ or *NRC2*/*3*/*4*‐silenced *Nicotiana* *benthamiana*. *Agrobacteria* carrying each clone were infiltrated 3 wk after virus‐induced gene silencing (VIGS). The *NRC2/3/4*‐dependent resistance gene *R8* and *NRG1*‐dependent resistance gene *N* were used as helper‐dependent controls. Each experiment was replicated independently twice (*n* < 10). All leaves were photographed at 3 d post infiltration (dpi). (b) Degree of cell death quantified as quantum yield (*F*_v_/*F*_m_) using a closed FluorCam system. Significance was determined using one‐way ANOVA followed by Dunnett’s multiple comparisons test, with asterisks denoting statistically significant differences (***, *P* < 0.0001). Data are mean values (± SD) of four biological replicates. (c) Relative transcript abundance for each gene was determined by quantitative reverse transcription‐polymerase chain reaction (RT‐PCR) analysis of silenced plants 3 wk after VIGS. The mean values for transcript levels were normalized to that of *N. benthamiana EF1‐α*. Transcript levels of GFP‐silenced plants were set to 1. Error bars represent SD of three biological replicates, and asterisks denote significant differences at ***, *P* < 0.0001, as determined by two‐way ANOVA followed by Sidak’s multiple comparisons test.

Recent studies have revealed that NLR‐mediated immunity involves a complex network, and that many NLRs require helper NLRs to exert activity in the immune response (Wu *et al*., 2017; Castel *et al*., 2019). We therefore tested the dependence of ANL‐ and CC^A^‐mediated cell death activity on known helper NLRs. Virus‐induced gene silencing was performed to simultaneously co‐silence the RNL‐type helpers *NbNRG1* and *NbADR1* and the triple NRC‐type helpers *NbNRC2*, *NbNRC3* and *NbNRC4*. Autoactive ANLs were expressed in silenced plants using agroinfiltration (Fig. 5a). Silencing efficiency was estimated by qRT‐PCR analysis of the transcript levels of each gene (Fig. 5c). *NRC*‐dependent *Phytophthora infestans* resistance gene, *R8* and *NRG1*‐dependent tobacco mosaic virus resistance gene, *N* were used as controls (Wu *et al*., 2017). Although cell death mediated by *R8* or *N* was compromised in *NRC*‐ and *NRG1/ADR1*‐silenced plants, respectively, silencing of these genes did not affect cell death mediated by *Pvr4*, autoactive ANLs (ANL620, ANL287 and ANL10‐2) or autoactive CC^A^309 (Fig. 5a,b). Moreover, *PR1* transcripts were sustained in these helper NLR‐silenced plants, suggesting that they are not required for ANL‐mediated resistance (Fig. S8). These results indicate that CC^A^s and ANLs may induce cell death and resistance in an RNL or NRC type helper‐independent manner.

### ANLs are conserved in seed plants, and CC^A^s of other plant species induce cell death

**Fig. 6:**
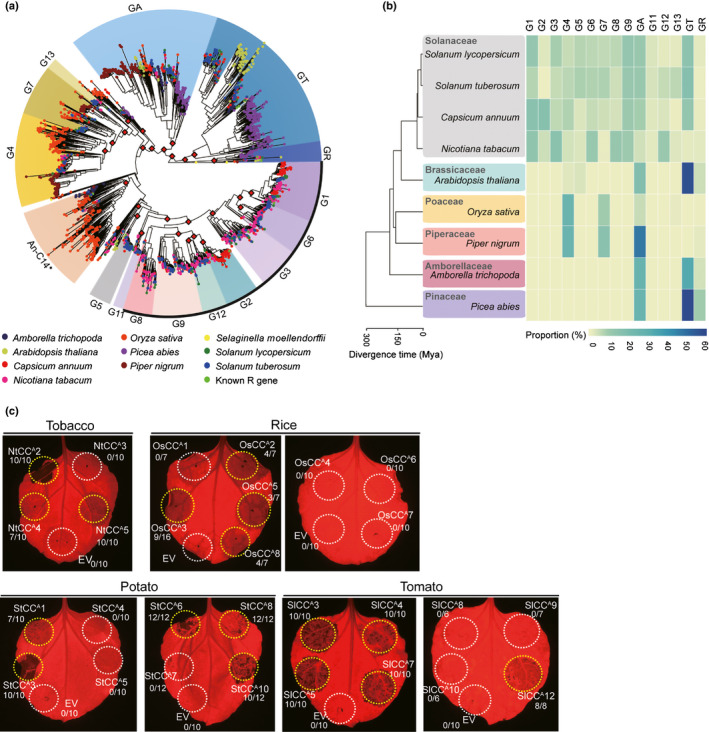
ANLs are conserved across seed plants, and CC^A^s of other plant species also induce cell death. (a) Intact NB‐ARC domains in NLRs from 10 plant genomes were used to reconstruct the phylogenetic tree. Groups were assigned based on ultrafast bootstrap (UFBoot) values guided by classification of Solanaceae NLRs. Two NB‐ARCs in *Selaginella* *moellendorffii* were used as the outgroup. UFBoot values > 90% are marked as red diamonds at the nodes. Black outline indicates NRC helper‐dependent groups. Asterisk denotes the clade described as An‐C14 in Shao et al. (2016). GA, group of ANLs; GR, group of RNLs. (b) The species tree (left) of nine plant genomes used in this study indicates their evolutionary relationships and divergence time. The proportion of NLRs of each group in plant species can be visualized using a heatmap (right). (c) CC^A^s from Solanaceae plants (potato, tomato, and tobacco) and a monocot plant (rice) were expressed in *Nicotiana* *benthamiana*. Autoactive cell death is indicated by yellow circles, and no cell death is indicated by white circles. The frequency of cell death induced by each CC^A^ is given in the form of a fraction. Images were acquired at 3 d post infiltration (dpi). The experiments were repeated three times with at least six biological replicates.

Phylogenetic analysis was conducted on a larger scale to more completely elucidate the divergence and evolutionary history of NLRs in seed plants. Because it is the only conserved domain of NLR proteins suitable for sequence alignment, the NB‐ARC domain of a total 2419 NLRs from ten representative plant species – including four Solanaceous species (pepper, tomato, potato, and tobacco (*N. tabacum*)); a Brassicaceae plant (Arabidopsis); a monocot, Poaceae rice (*O. sativa*); a Piperaceae plant, black pepper (*P. nigrum*), a basal angiosperm (*Amborella trichopoda*); a gymnosperm, Norway spruce (*P. abies*) and one of the oldest lineages of vascular plants *Selaginella* (*Selaginella moellendorffii*) – were subjected to phylogenetic analysis. The groups were assigned based on a previous study of the classification of Solanaceae NLRs (Seo *et al*., 2016). Most of the NLRs were included in assigned groups, except for three NLRs of *Selaginella*. The ANLs formed a monophyletic clade with a high bootstrap value of 91% which was clearly distinguishable from the other CNL clades, despite being typical CNLs (Fig. 6a). We also found that nine of ten species (except *Selaginella*) possess this ANL clade. To determine whether this ANL clade is unique among a wide variety of species, we analyzed the NLR repertoires for each plant species based on their clustering. Interestingly, ANLs were present in all of the plant species examined, even in the most ancestral angiosperm and gymnosperm plants (Fig. 6b; Dataset S4). These results suggest that ANLs are distinct from other clades of CNLs and have existed since the emergence of seed plants.

Next, we examined the commonality of CC^A^‐mediated cell death among other plants. Representative CC^A^s from five plant species (pepper, tomato, potato, tobacco, and rice) were randomly chosen from a subclade in the phylogenetic tree (Dataset S1). More than 40% of tested CC^A^s from Solanaceae plants, and even taxonomically distant rice, trigger cell death in *N. benthamiana* (Fig. 6c; Table S3). Protein accumulation was examined by immunoblot analysis (Fig. S9). In addition, a recent study reported that a high portion of the predicted CC domain of the CNLs in Arabidopsis ecotype Columbia‐0 triggered cell death (Wroblewski *et al*., 2018). According to our classification, all of the CNLs from group B described by Wroblewski *et al.,* (2018), except At1g63360, belonged to the ANL group, and 13 of 22 CC^A^ domain fragments induced cell death in *N. benthamiana*. Together with this previous report, our results indicate that the CC^A^s from dicot Solanaceae plants as well as the monocot rice plant trigger cell death.

## Discussion

NLRs are one of the largest and most widespread families of intracellular immune receptors in plants. These proteins activate potent immune responses that often accompany a HR. We demonstrated that among the NLR groups in pepper, the ANL is noteworthy as one of the NLR group with an autoactive coiled‐coil domain. Autoactive CC^A^‐induced cell death resembles R gene‐mediated HR and immune responses, suggesting that ANLs can trigger immune signaling. Analyses of pepper CC^A^ sequences revealed that nonautoactive CC^A^s contain partial deletions or variations in the α1 helix, and that the α1 helix is critical for cell death‐inducing activity. Furthermore, cell death mediated by ANLs is independent of RNLs or NRC type helper NLRs, and ANLs are widely conserved in seed plants.

In this study, nearly all (436) of the full‐length NLRs in the pepper genome were systemically tested for cell death‐inducing activity in *N. benthamiana*. It was shown that only 15 of these NLRs exhibit autoactivity, and they are distributed across various groups, such as TNL, CNL‐G5, G9, G10 (ANL), G11 and NG (Table 1). Of the 15 autoactive NLRs examined, only 5 NTD domains from autoactive NLRs induced visible cell death (Table S2). Intriguingly, the other 10 NLRs harboring a nonautoactive NTD also triggered cell death. These results suggest that other domain(s) may be responsible for autoactivity such as *Tomato spotted wilt virus* resistant protein Sw‐5b, and Arabidopsis RPS5 (Ade *et al*., 2007; De Oliveira *et al*., 2016). Sw‐5b, a CNL type R protein, triggers cell death in the absence of its cognate effector, NSm (Brommonschenkel *et al*., 2000). Only the NB‐ARC domain of Sw‐5b is sufficient to induce cell death – the NTD alone is not (De Oliveira *et al*., 2016). Arabidopsis RPS5, and CC‐NB‐ARC but not CC alone are able to induce cell death (Ade *et al*., 2007).

Pepper ANLs fall into their own monophyletic group containing a high proportion of autoactive CC domains, and three out of 33 ANLs also showed autoactivity (Table 1). These lead us to assume that ANLs have unique feature(s) in terms of NLR‐mediated cell death. We therefore examined whether they function as executer NLRs in typical NLR pairs. It has been shown that executor NLRs are functionally coupled to sensor NLRs. Sensor NLRs often contain additional nonconserved domains that function in pathogen recognition and are coupled to an executor NLR to initiate immune signaling (Rodriguez‐Moreno *et al*., 2017). In the case of typical paired NLRs, sensor NLRs form heterodimers with executor NLRs to suppress autoactivity in the absence of pathogen detection (Jin *et al*., 2002; Zhang *et al*., 2004). The recognition of pathogen by a sensor NLR leads to a change in the executor NLR state from inactive to active. Generally, typical NLR pairs are genetically linked and oriented head‐to‐head, sharing a promoter for simultaneous regulation of transcription (Narusaka *et al*., 2009; Saucet *et al*., 2015). We found that autoactive pepper ANLs are not oriented head‐to‐head in the neighboring region and that no NLRs contain the additional integrated domain (Table S1). Additional genome‐wide analysis using updated pseudomolecules of pepper revealed that 19 NLR pairs exist in head‐to‐head orientation in pepper. Only one ANL (referred to as CaNBARC403) was identified; however, CaNBARC403 does not have autoactivity or an additional domain on its upstream flanking gene (Table S4). These data suggest that autoactive ANLs in pepper do not function as executors in typical NLR pairs, and their autoactivity might be regulated by proper transcription or negative regulatory protein(s), such as RIN4, which negatively regulates RPS2 (Day *et al*., 2005).

Recently, singleton NLR was defined to function as single genetic unit for sensing and signaling (Adachi *et al*., 2019b). Behaving both as a sensor and executor, singleton NLRs are thought not to require helper NLRs, implying that singleton NLRs may be functional when transferred to taxonomically distinct plant species in the absence of their helper NLRs (Adachi *et al*., 2019b). Based on several lines of evidence, we propose that pepper ANLs might be classified as putative singleton NLRs. First, many CC^A^s from various plant species exhibit autoactivity (Tables 1, S3; Fig. 6). In addition, a recent study reported that 9 of 22 Arabidopsis CNLs in group B that are classified as ANLs in our study trigger cell death in Arabidopsis (Wroblewski *et al*., 2018). These results indicate that ANLs have the potential to initiate signaling via their autoactive CC domain. Second, a functional analysis of ANL‐R proteins revealed that they specifically sense cognate effector proteins, as shown by Pvr4 recognizing PepMoV‐NIb (Kim *et al*., 2018) and RPS5 recognizing AvrPphB (Ade *et al*., 2007). Taken together, these data indicate that ANLs might sense effectors directly or indirectly as well as initiating immune signaling. Finally, ANLs may function independently of helper NLRs. Phylogenetic tree analysis indicates that ANLs are distinct from the NRC‐dependent clade of NLRs (Fig. 6a). As expected, ANL‐mediated cell death showed sustainable cell death in *NRC*‐silenced plants (Fig. 5a). Additionally, we observed that cell death was also not compromised in RNL‐type helper‐silenced plants (Fig. 5a). This helper‐independent signaling facilitates that CC^A^s also trigger visible cell death in taxonomically distant plants. However, it is doubt that all ANLs across plant species share same mechanism to confer resistance. Arabidopsis *ADR1* and *NRG1* are completely or partially required for *RPS2*‐mediated resistance (Bonardi *et al*., 2011).

We also demonstrated that CC^A^s of monocot rice induce cell death in dicot *N. benthamiana* (Fig. 6c; Table S3). In addition, of 22 Arabidopsis CC^A^s, 13 have been shown to induce cell death in *N. benthamiana*, and 5 in lettuce (Wroblewski *et al*., 2018). Therefore, we conclude that ANLs may not require known helper NLR for their autoactivity and ANL‐mediated cell death is well conserved over a wide range of plant species.

Adachi *et al*. recently suggested that NLRs evolved from multifunctional singleton receptors to functionally specialized and diversified receptor pairs and intricate receptor networks (Adachi *et al*., 2019b). We demonstrated that ANLs form a distinct phylogenetic cluster that includes several cloned functional R genes: *Pvr4* and *Tsw* in pepper and *RPS2* and *RPS5* in Arabidopsis (Fig. 6a; Dataset S4). We also demonstrated that ANLs are well conserved across multiple plant species, including the basal angiosperm *A. trichopoda* and the gymnosperm *P. abies* (Fig. 6b; Table S5). A previous study also reported that the AN‐C2 group corresponding to an ANL group is an ancestral angiosperm lineage, suggesting that ANLs are ancient compared with other CNL groups (Shao *et al*., 2016).

In the point mutation studies on CC^A^309, only the mutation at putative phosphorylation sites T7A and S11A enhanced cell death compared with wild‐type CC^A^309. Surprisingly CC^A^309 induced cell death is significantly diminished in phospho‐mimic mutants (Fig. 4d). However, the mechanism by which phosphorylation/dephosphorylation upon activation of NLR protein triggers cell death has remained obscure. It would be interesting to confirm in the future whether the activation of NLRs is indeed regulated by phosphorylation/dephosphorylation events. We have also shown that CC^A^309 induced cell death is inhibited by a calcium chelating agent and a Ca^2+^ influx blocker (EGTA and LaCl_3_). Ca^2+^ is a well conserved second messenger in most aspects of cellular signaling programs. Ca^2+^ influx is known to be required for cell death triggered by RPS2/AvrRpt2 interactions (Gao *et al*., 2013). Taken together, our findings indicate that ANL‐mediated cell death involves the influx of calcium ions into the cytoplasm.

Analyses of pepper CC^A^ sequences indicated the presence of partial deletions or variations in the α1 helix region in nonautoactive CCs (Fig. 2). Mutations in the α1 helix of CC^A^s or ANL compromised their cell death‐inducing activity (Fig. 3). This region is matched with the ZAR1 α1, forming a funnel‐shaped structure in the PM after conformational switching during activation of the ZAR1 resistosome, and amphipathic residues in the ZAR1 α1 helix are known to be essential for cell death‐inducing activity (Adachi *et al*., 2019a; Wang *et al*., 2019a). Collectively, these data indicate that the α1 helix is important for triggering cell death in singleton NLRs. It would be interesting to examine in future experiments whether pepper CC^A^s or ANLs form multimers similar to the ZAR1 resistosome in order to elucidate the general mechanism of NLR‐induced cell death. Here, we suggest that ANLs not only serve as valuable resources for increasing our understanding of the molecular basis of NLR‐mediated cell death but also provide critical information for identifying new disease‐resistance genes in crop plants.

## Author contributions

DC conceived the project; HL, HM and DC designed the experiments; HL, HM, EC, YS, SO and SK performed the experiments; MK analyzed the data; and HL, HM, MK and DC wrote the manuscript.

## Supporting information


**Dataset S1** List of primers used for polymerase chain reaction (PCR) and quantitative reverse transcription‐PCR.
**Dataset S2** The sequence of fragments for screening of autoactive pepper nucleotide‐binding leucine‐rich repeats (NLRs).
**Dataset S3** The sequences of cloned N‐terminal domains (NTDs).
**Dataset S4** The NLR genes used for phylogenetic analysis in this study.

**Fig. S1** Cell death induced by pepper autoactive coiled‐coil domains (CC^A^s) in *Nicotiana benthamiana*.
**Fig. S2** Prediction of EDVID or MADA motif in CC domains in pepper NLRs
**Fig. S3** Cell death phenotype of green fluorescent protein (GFP)‐tagged CC^A^309 and CC^A^10‐1.
**Fig. S4** No visible cell death in the leaves expressing CC^A^s and ancient and autonomous NLRs (ANLs) with pCAMBIA2300:GFP or PVX:GFP.
**Fig. S5** Alignment of amino acid sequences of pepper CC^A^s.
**Fig. S6** CC^A^309 and CC^A^10‐1 localize to the plasma membrane.
**Fig. S7** CC^A^s in subclades I and II also localize to the plasma membrane regardless of their cell death‐inducing activity.
**Fig. S8** The level of *PR1* transcripts in NLR‐or CC^A^‐expressing cells in known helper NLR‐silenced plants.
**Fig. S9** The protein expression level of CC^A^s from tobacco, rice, potato and tomato.
**Table S1** Gene orientation and predicted function of an upstream gene of an autoactive pepper NLR.
**Table S2** List of autoactive NLRs and autoactive N‐terminal domains in pepper.
**Table S3** CC^A^s of other Solanaceae plants as well as rice and Arabidopsis are also capable of inducing cell death in *N. benthamiana*.
**Table S4** Identification of head‐to‐head oriented NLR genes in pepper genome.
**Table S5** The number of NLRs for each group in ten plant genomes.
Please note: Wiley Blackwell are not responsible for the content or functionality of any Supporting Information supplied by the authors. Any queries (other than missing material) should be directed to the *New Phytologist* Central Office.
