# Supplementary material for: Genome‐wide functional analysis of hot pepper immune receptors reveals an autonomous NLR clade in seed plants
Source: New Phytol. 2020 Nov 16;229(1):532–47. doi: 10.1111/nph.16878 (PMC7756659; doi:10.1111/nph.16878)
Supplement: Supplementary file 2 — Fig. S1 Cell death induced by pepper autoactive coiled‐coil domains (CCAs) in Nicotiana benthamiana. Fig. S2 Prediction of EDVID or MADA motif in CC domains in pepper NLRs Fig. S3 Cell death phenotype of green fluorescent protein (GFP)‐tagged CCA309 and CCA10‐1. Fig. S4 No visible cell death in the leaves expressing CCAs and ancient and autonomous NLRs (ANLs) with pCAMBIA2300:GFP or PVX:GFP. Fig. S5 Alignment of amino acid sequences of pepper CCAs. Fig. S6 CCA309 and CCA10‐1 localize to the plasma membrane. Fig. S7 CCAs in subclades I and II also localize to the plasma membrane regardless of their cell death‐inducing activity. Fig. S8 The level of PR1 transcripts in NLR‐or CCA‐expressing cells in known helper NLR‐silenced plants. Fig. S9 The protein expression level of CCAs from tobacco, rice, potato and tomato. Table S1 Gene orientation and predicted function of an upstream gene of an autoactive pepper NLR. Table S2 List of autoactive NLRs and autoactive N‐terminal domains in pepper. Table S3 CCAs of other Solanaceae plants as well as rice and Arabidopsis are also capable of inducing cell death in N. benthamiana. Table S4 Identification of head‐to‐head oriented NLR genes in pepper genome. Table S5 The number of NLRs for each group in ten plant genomes. Please note: Wiley Blackwell are not responsible for the content or functionality of any Supporting Information supplied by the authors. Any queries (other than missing material) should be directed to the New Phytologist Central Office. [file NPH-229-532-s002.pdf]

New Phytologist

Article title: Genome-wide functional analysis of hot pepper immune receptors reveals an autonomous NLR cluster in seed plants

Authors: Hye-Young Lee, Hyunggon Mang, Eun-Hye Choi, Ye-Eun Seo, Myung-Shin Kim, Soohyun Oh, Saet-Byul Kim, and Doil Choi

Article acceptance date: 07 August 2020

Table S1 Gene orientation and predicted function of an upstream gene of an autoactive pepper NLR

Autoactive NLR		Upstream gene		
Gene ID (NLR ID)	Strand	Gene ID	Strand	Gene description
CA11g06220 (NLR502)	+	CA11g06230	+	Partial TNL-type protein
CA12g06200 (NLR504)	+	CA12g06190	-	Putative nuclease HARBI1-like
CA12g16200 (NLR582)	+	CA00g70880	-	Ribosomal protein S11
CA12g19860 (NLR530)	+	CA12g19850	+	Partial TNL-type protein
CA12g19870 (NLRT2)	+	CA12g19860	+	NLR530 (Autoactive TNL)
CA12g19890 (NLRT1)	+	CA12g19880	+	Partial TNL-type protein
CA05g05510 (NLR110)	+	CA05g05520	-	Putative LRR receptor-like serine/threonine-protein kinase
CA05g02490 (NLR5)	+	CA05g02480	+	Partial CNL-type protein (CNL-NG)
CA01g29850 (NLR287)	+	CA01g29860	+	CNL-type protein (CNL-NG)
CA01g33790 (NLR620)	+	CA01g33800	+	CNL-type protein (G10)
CA01g33850 (NLR10-2)	+	CA01g33840	+	CNL-type protein (G10)
CA07g09720 (NLR174)	+	CA07g09730	+	Pentatricopeptide repeat-containing protein
CA07g12630 (NLR168)	+	CA07g12640	+	CNL-type protein (CNL-NG)
CA08g02000 (NLR95)	+	CA08g01990	+	Partial NL-type protein
CA12g19770 (NLR179)	+	CA12g19760	+	Phloem protein 2-like protein

Table S2 List of autoactive NLRs and autoactive N-terminal domains in pepper

Group	Full-length NLR (NLR ID)	N-terminal domain (NTD ID)
TNL	CA11g06220 (NLR502)	CA12g19860 (TIR530)*
	CA12g06200 (NLR504)	CA12g19870 (TIRT2)*
	CA12g16200 (NLR582)	
	CA12g19860 (NLR530)*	
	CA12g19870 (NLRT2)*	
	CA12g19890 (NLRT1)	
CNL-G5	CA05g05510 (NLR110)	
CNL-G9	CA05g02490 (NLR5)	
CNL-ANL	CA01g29850 (NLR287)*	CA00g76890 (CC403)
	CA01g33790 (NLR620)*	CA01g29850 (CC287)*
	CA01g33850 (NLR10-2)*	CA01g33790 (CC620)*
		CA01g33850 (CC10-2)*
		CA01g33880 (CC327)
		CA01g33900 (CC466)
		CA04g10720 (CC585)
		CA09g17050 (CC380)
		CA09g17080 (CC436)
		CA09g17090 (CC773)
		CA10g20540 (CC328)
		CA10g20630 (CC333)
		CA10g20820 (CC223)
		CA10g21090 (CC398)
		CA10g21120 (CC309)
		CA10g21170 (CC322)
		CA10g21380 (CC516)
CNL-G11	CA07g09720 (NLR174)	CA03g00800 (CC281)
CNL-RNL		CA02g25800 (RPW595)
		CA04g19370 (RPW405)
CNL-NG	CA07g12630 (NLR168)	
	CA08g02000 (NLR95)	
	CA12g19770 (NLR179)	

*: The gene that both full length NLR and their N-terminal domain show autoactivity in *Nicotiana benthamiana*

Table S3 CC^As of other Solanaceae plants as well as rice and *Arabidopsis* are also capable of inducing cell death in *N. benthamiana*.

	Assigned ANLs	Tested CC^A	Autoactive CC^A domain (%)
Pepper	34	24	17 (70.8)
Potato	32	8	5 (62.5)
Tomato	15	8	5 (62.5)
Tobacco	8	4	3 (75)
Rice	13	8	4 (50)
Arabidopsis*	23	22	13 (59.1)

* This result is quote from Wróblewski et al., (2019).

New Phytologist

Table S4 Identification of head-to-head oriented NLR genes in pepper genome.

Gene ID	Type[a]	Group[b]	Autoactivity[c]	Integrated domain	Upstream gene				
					Gene ID	Type[a]	Group[b]	Autoactivity[c]	Integrated domain
CA.PGAv.1.6.scaffold137.47	N	N.A.	N.D		CA.PGAv.1.6.scaffold137.46	N	N.A.	N.D	
CA.PGAv.1.6.scaffold282.124	CNL	G9	N		CA.PGAv.1.6.scaffold282.123	CNL	G9	N	
CA.PGAv.1.6.scaffold309.40	CNL	G4	N		CA.PGAv.1.6.scaffold309.39	CNL	G4	N	
CA.PGAv.1.6.scaffold551.100	CN	N.A.	N.D		CA.PGAv.1.6.scaffold551.99	CNL	G2	N	
CA.PGAv.1.6.scaffold676.64	CNL	G7	N		CA.PGAv.1.6.scaffold676.63	N	N.A.	N.D	
CA.PGAv.1.6.scaffold767.32	CNL	G3	N	SD	CA.PGAv.1.6.scaffold767.31	CNL	G3	N	SD
CA.PGAv.1.6.scaffold1084.23	XN	N.A.	N.D	NACK_C	CA.PGAv.1.6.scaffold1084.22	CNL	G2	N	
CA.PGAv.1.6.scaffold1090.4	CNL	G2	N		CA.PGAv.1.6.scaffold1090.3	CNL	G2	N	
CA.PGAv.1.6.scaffold1090.13	N	N.A.	N		CA.PGAv.1.6.scaffold1090.11	CNL	G2	N	
CA.PGAv.1.6.scaffold1090.34	CNL	G12	N		CA.PGAv.1.6.scaffold1090.33	CNL	G2	N	
CA.PGAv.1.6.scaffold1090.37	XN	N.A.	N.D	Haem-oxygenase	CA.PGAv.1.6.scaffold1090.36	N	N.A.	N	
CA.PGAv.1.6.scaffold1357.10	CNL	G2	N		CA.PGAv.1.6.scaffold1357.9	CNL	G2	N	
CA.PGAv.1.6.scaffold1357.16	CNL	G2	N		CA.PGAv.1.6.scaffold1357.15	CNL	G2	N	
CA.PGAv.1.6.scaffold1373.15	CNL	G2	N.D		CA.PGAv.1.6.scaffold1373.14	N	N.A.	N.D	
CA.PGAv.1.6.scaffold1578.13	CNL	G2	N		CA.PGAv.1.6.scaffold1578.12	CNL	G2	N	
CA.PGAv.1.6.scaffold1640.8	CN	N.A.	N.D		CA.PGAv.1.6.scaffold1640.7	CNL	G2	N	
CA.PGAv.1.6.scaffold1646.8	ANL	ANL	N		CA.PGAv.1.6.scaffold1646.7	CNL	G1	N	
CA.PGAv.1.6.scaffold1677.2	N	N.A.	N		CA.PGAv.1.6.scaffold1677.1	XN	N.A.	N.D	NACK_C
CA.PGAv.1.6.scaffold1982.2	NL	N.A.	N.D		CA.PGAv.1.6.scaffold1982.1	N	N.A.	N.D	

[a]NLR type: CNL, CC-NBARC-LRR; ANL, ancient and autonomous NLR; N, NBARC; CN, CC-NBARC; NL, NBARC-LRR; XN, extra domain-NBARC
[b]Group: N.A., Not assigned; N, No autoactivity
[c]Autoactivity: N.D, Not determined; N, No autoactivity

New Phytologist

Table S5 The number of NLRs for each group in ten plant genome.

	G1	G2	G3	G4	G5	G6	G7	G8	G9	G10 (GA)	G11	G12	G13	GT	GR	NG	Total
Solanum lycopersicum	21	3	14	8	11	10	9	10	20	18	4	2	2	25	2	12	171
Solanum tuberosum	20	0	15	18	23	21	22	12	33	32	9	15	17	52	1	14	304
Capsicum annuum	67	69	23	29	9	20	16	13	41	35	7	10	1	52	3	23	418
Nicotiana tabacum	48	15	56	12	8	40	2	42	36	13	5	38	7	4	5	39	370
Arabidopsis thaliana	0	0	0	2	3	0	0	0	0	23	0	0	0	73	5	18	124
Oryza sativa	0	0	0	98	0	0	34	0	0	17	0	0	3	0	1	222	375
Piper nigrum	0	0	0	80	0	0	49	0	0	150	0	0	0	0	3	26	308
Amborella trichopoda	0	0	0	0	0	0	0	0	0	17	0	0	0	21	2	23	63
Picea abies	0	0	0	0	0	0	0	0	0	92	0	0	0	208	31	15	346
Selaginella moellendorffii	0	0	0	0	0	0	0	0	0	0	0	0	0	0	0	3	3

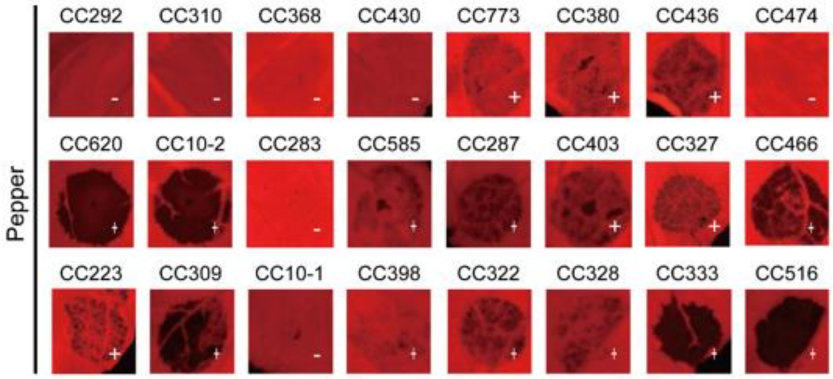

Fig. S1 Cell death induced by pepper CC^{A}s in *N. benthamiana*. 24 CC^{A} of pepper ANLs were transiently overexpressed in *N. benthamiana*. Images were taken at 3 days after infiltration. Cell death and no visible cell death are presented + and -, respectively. Experiments were repeated three times. n=10.

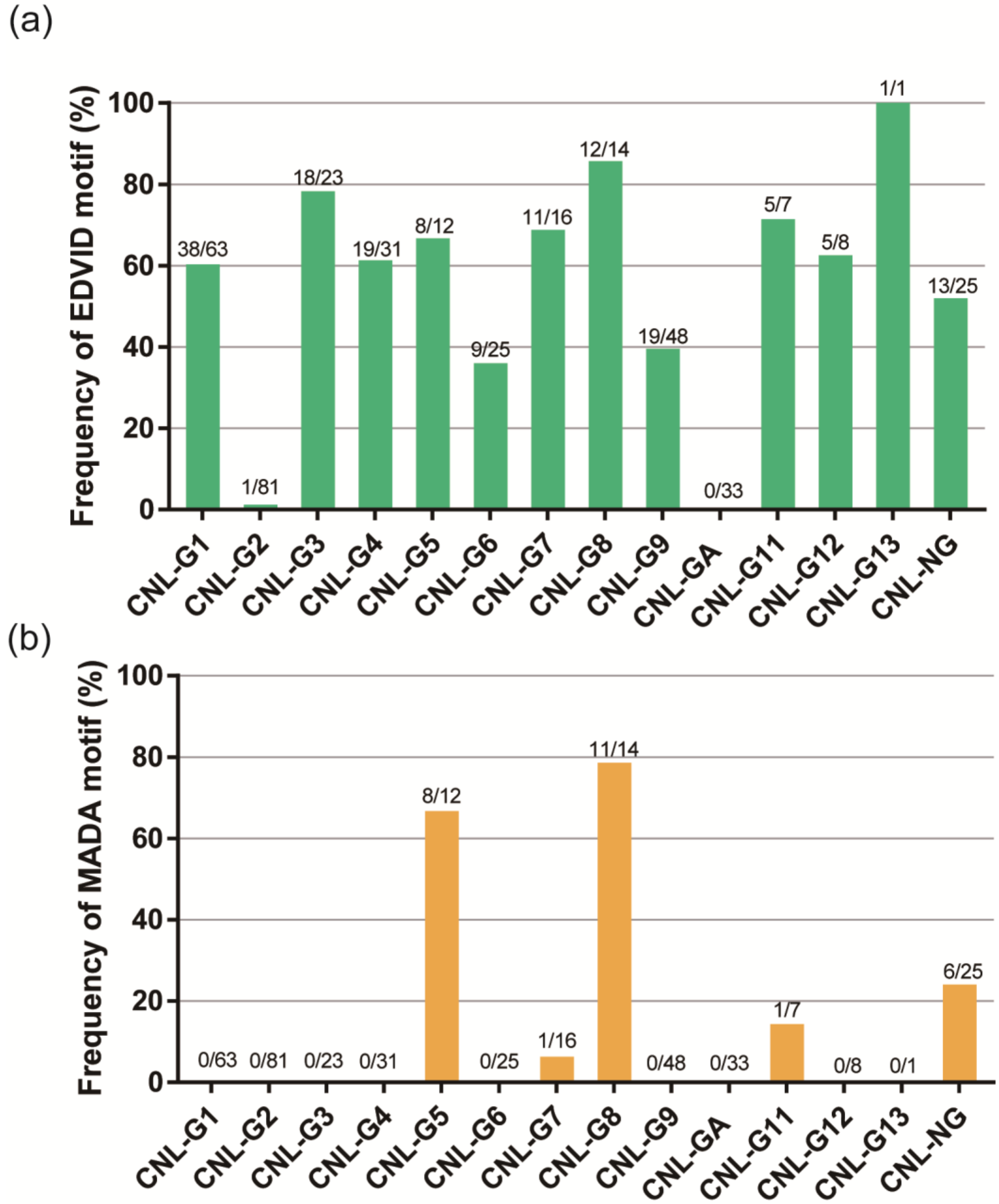

Fig. S2 Prediction of EDVID or MADA motif in CC domains in pepper NLRs The frequency of EDVID motif (a) and MADA motif (b) in CC domains for each groups. EDVID motif (motif_16 from NLR-Parser) and MADA-HMM from Adachi et al., 2019 were used to scan pepper NLRs. An HMM score cut-off was followed by Adachi *et al*., 2019.

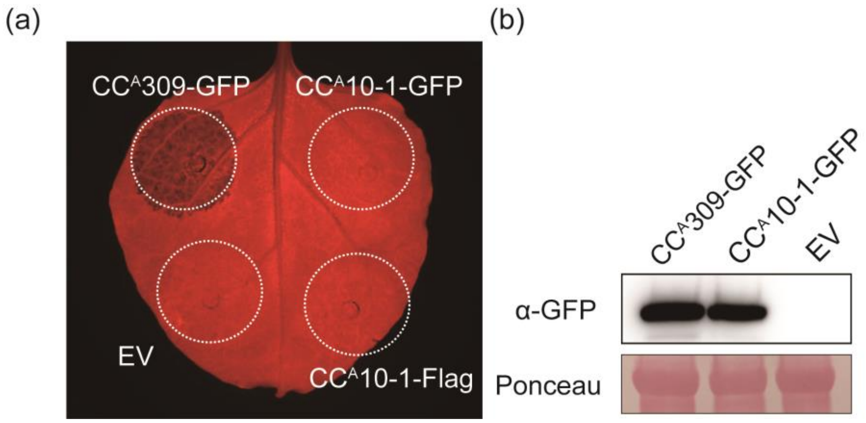

Fig. S3 Cell death phenotype of GFP-tagged CC^A309 and CC^A10-1. (a) CC^A309-GFP and CC^A10-1-GFP were expressed in *N. benthamiana* leaves by Agroinfiltration with an empty vector (EV) as a negative control. Images were taken 2 days post infiltration. (b) Protein accumulation was examined by western blot analysis. Equal protein loading was confirmed by membrane staining by ponceau solution.

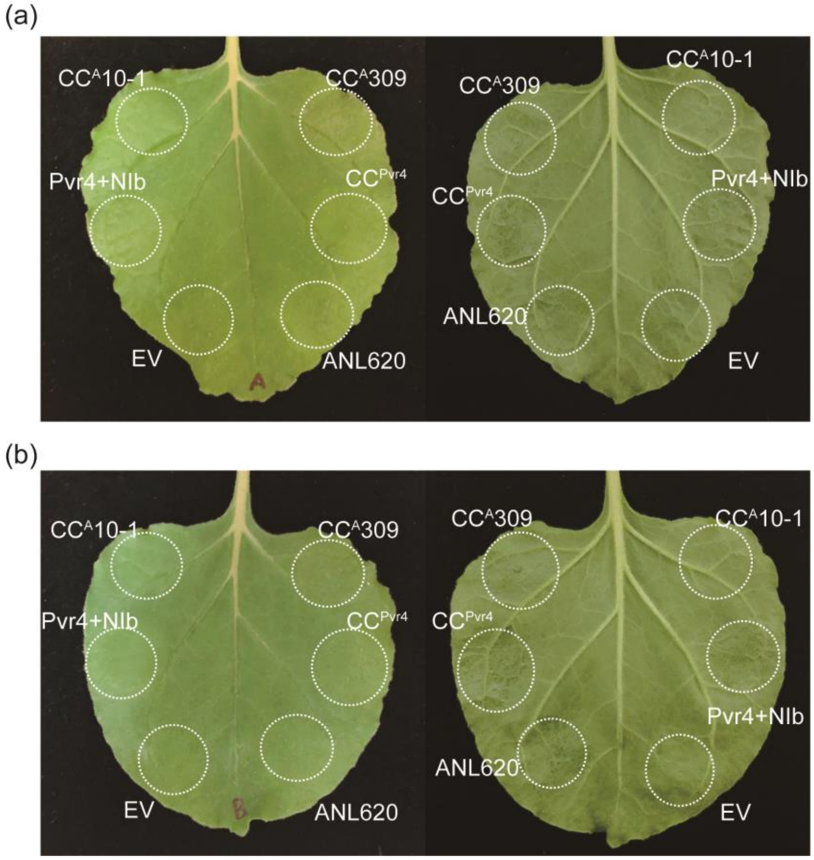

Fig. S4 No visible cell death in the leaves expressing CC^{A}s and ANLs with pCAMBIA2300:GFP or PVX:GFP. Leaves were infiltrated pCAMBIA2300:GFP (a) or PVX:GFP (b) with ANLs or CC^{A}s. Right panel represents adaxial side and left panel shows abaxial side. Same leaves used in Figure 1e were photographed under white light condition 30 hr after agroinfiltration. n=8.

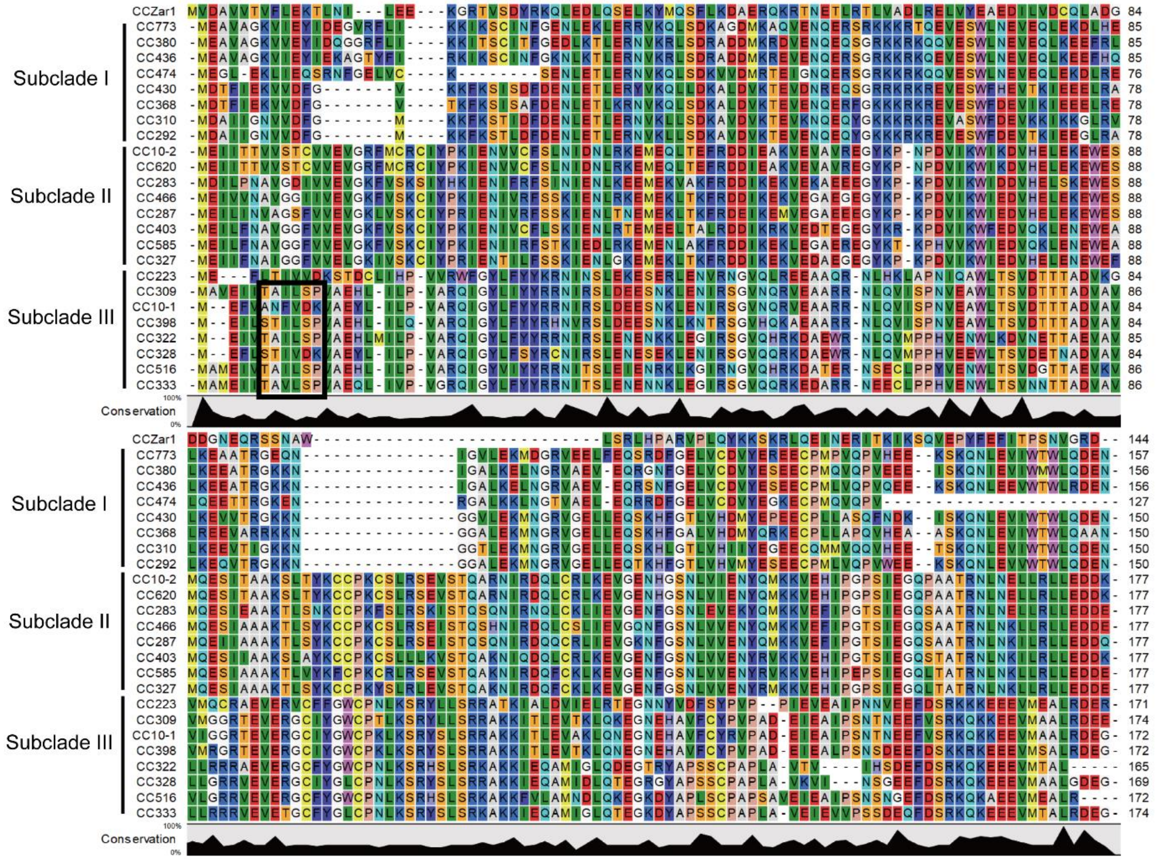

Fig. S5 Alignment of amino acid sequences of pepper CC^{A}s. Amino acid sequences of 24 pepper CC^{A}s and the CC domain of *Arabidopsis* ZAR1 were used for sequence alignment. Alignment were conducted by CLC workbench software. The conserved motif (TAILSP) in clade III was presented in a black box.

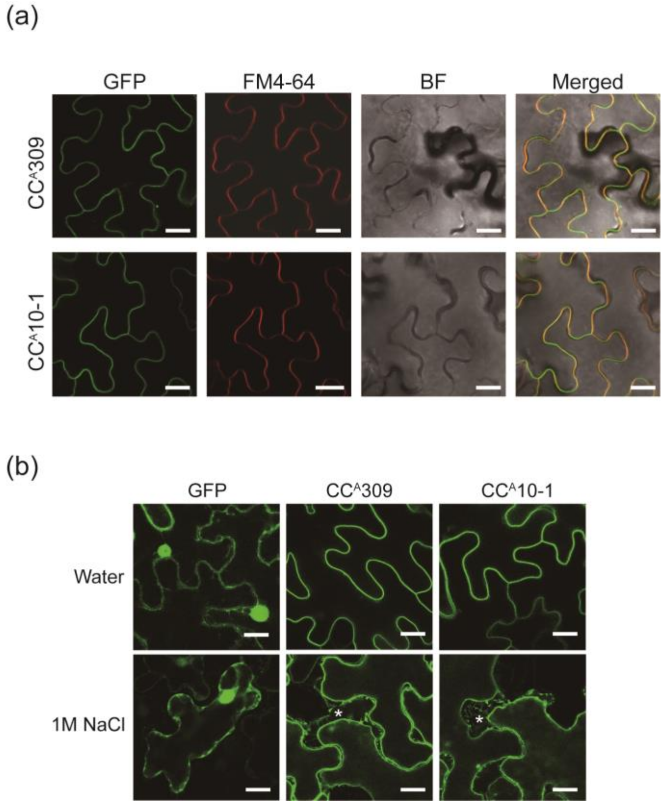

Fig. S6 CC^{A}309 and CC^{A}10-1 localize to plasma membrane. (a) Co-localization of CC^{A}309-GFP and CC^{A}10-1-GFP with FM 4-64–labeled PM. Confocal microscopy images were taken at 26 hpi; bar = 20 μm. (b) Plasmolyzed cells by 1M NaCl solution. Asterisks indicate Hechtian strands.

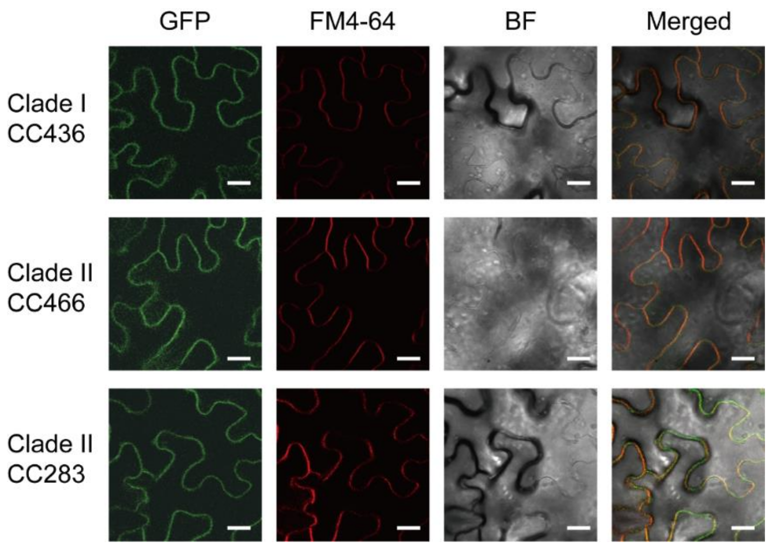

Fig. S7 CC^{A}s in subclade I and II also localize at plasma membrane regardless of their cell death inducing activity. Autoactive CC^{A}436 in subclade I, autoactive CC^{A}466 in subclade II and non-autoactive CC^{A}283 in subclade II were chosen to explore their subcellular localization. GFP was fused to these proteins at C-terminus and GFP-fused proteins were expressed in *N. benthamiana*. GFP signal was observed by confocal microscopy at 48 hr after infiltration. FM 4-64 dye was used as a PM-marker fluorescence dye. Bar=20 μm.

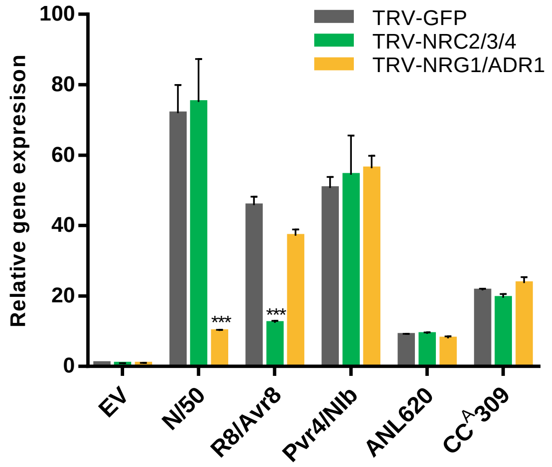

Fig. S8 The level of *PR1* transcripts in NLR-or CC^{A}-expressing cells in known helper NLR-silenced plants. Expression level of *PR1* was measured by qRT-PCR in leaf tissue infiltrated *Agrobacteria* carrying ANLs (Pvr4, ANL620) or CC^{A} (CC^{A}309). Leaf tissue was collected at 36 hpi and RNA was extracted from collected tissue. N/p50 and R8/Avr8 were used for *NRG1*- or *NRCs*-dependent resistance gene, respectively. Significance was determined using one-way ANOVA followed by Tukey's multiple comparisons test, with asterisks denoting statistically significant differences: ***P < 0.0001. Data are the mean (± SD) of four biological replicates. Error bars indicate standard deviation.

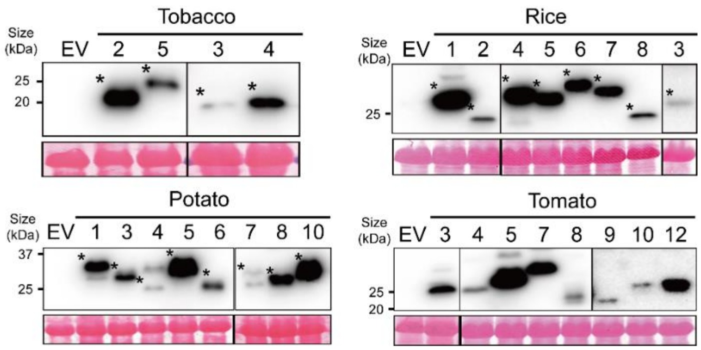

Fig. S9 The protein expression level of CC^{A}s from tobacco, rice, potato and tomato Protein accumulation were analyzed by immunoblot. Asterisks indicate the expected band of proteins. Equal protein loading was confirmed by Ponceau S stained membrane.

References

Adachi H, Contreras M, Harant A, Wu C, Derevnina L, Sakai T, Duggan C, E. M, Bozkurt TO, Maqbool A, et al. 2019a. An N-terminal motif in NLR immune receptors is functionally conserved across distantly related plant species. *Elife* **8**. e49956.

Wroblewski T, Spiridon L, Martin EC, Petrescu AJ, Cavanaugh K, Truco MJ, Xu H, Gozdowski D, Pawlowski K, Michelmore RW, et al. 2018. Genome-wide functional analyses of plant coiled-coil NLR-type pathogen receptors reveal essential roles of their N-terminal domain in oligomerization, networking, and immunity. *PLoS Biology* **16**(12): e2005821.
